# Association between Healthy Eating Index-2015 and Breast Cancer Risk: A Case-Control Study

**DOI:** 10.31557/APJCP.2020.21.5.1363

**Published:** 2020-05

**Authors:** Hamed Kord- Varkaneh, Ammar Salehi- Sahlabadi, Meysam Zarezadeh, Jamal Rahmani, Shing Cheng Tan, Azita Hekmatdoost, Bahram Rashidkhani

**Affiliations:** 1 *Student Research Committee, Department of Clinical Nutrition and Dietetics, Faculty of Nutrition and Food Technology, Shahid Beheshti University of Medical Sciences, Tehran, Iran.*; 2 *Department of Clinical Nutrition, Student Research Committee, Nutrition Research Center, School of Nutrition and Food Sciences, Tabriz University of Medical Sciences, Tabriz, Iran. *; 3 *UKM Medical Molecular Biology Institute, Universiti Kebangsaan Malaysia, Kuala Lumpur, Malaysia.*; 4 *Department of Community Nutrition, Faculty of Nutrition Sciences and Food Technology, Shahid Beheshti University of Medical Sciences, Tehran, Iran. *

**Keywords:** Healthy Eating Index-2015, breast cancer, diet

## Abstract

**Objective:**

Diet quality is known to influence cancer risk. The Healthy Eating Index (HEI) is one of the most frequently used measures of diet quality. However, the association between HEI-2015 and breast cancer risk is not known. The present study was undertaken to evaluate the association between HEI-2015 and breast cancer risk.

**Methods:**

A case-control study comprising 134 breast cancer patients and 265 cancer-free controls were conducted. Dietary intakes were assessed using a validated food frequency questionnaire (FFQ), from which the HEI-2015 score was calculated. Logistic regression was used to derive the odds ratios (ORs) for measuring the association between HEI-2015 scores and breast cancer risk.

**Results:**

Subjects in the top quartile of HEI-2015 had a 46% lower chance of breast cancer compared with subjects in the bottom quartile (OR 0.54; 95% CI 0.30, 0.98). After adjustment for potential confounders such as age, age at menarche, oral contraceptive drug use, menopausal status, marital status, body mass index, smoking and education level, the association between HEI-2015 score and a lower risk of breast cancer was enhanced (OR 0.32; 95% CI 0.16, 0.65).

**Conclusion:**

We successfully demonstrated that a higher HEI-2015 score was associated with a reduced breast cancer risk.

## Introduction

Breast cancer (BC) is the most common type of cancer among women, both in terms of incidence and mortality (Edefonti et al., 2009; Fitzmaurice et al., 2017). In 2012 alone, approximately 1.7 million women were diagnosed with BC globally. This figure is expected to rise steadily to 3.2 million in year 2050 (Tao et al., 2015). Currently, the incidence of BC in western countries is higher than in Asian populations, but the rate is increasing at an alarming rate in Asian countries (Youlden et al., 2014). In Iran, the incidence of BC is about 22 cases per 100,000 women (Mousavi et al., 2007). 

Several risk factors have been unequivocally associated with cancer incidence, including non-modifiable factors such as genetics, age and gender, and modifiable factors such as the food and the diet (Perera, 1997; Meyerhardt et al., 2007; Brennan et al., 2010). For example, it has been shown that foods rich in phytochemicals and antioxidants, and low in saturated and trans fatty acids, have protective effects against cancers (Kushi and Giovannucci, 2002; Borek, 2004). More recently, a link between the quality of the overall diet and cancer risk has been suggested. The Healthy Eating Index (HEI) is one of the most commonly used measures of diet quality (Guenther et al., 2013). The HEI is updated every five years, with the most recent update made in year 2015 (HEI-2015). The HEI-2015 assesses diet quality based on the intake of 13 components, namely total fruits, whole fruits, total vegetables, greens and beans, whole grains, dairy, total protein foods, seafood and plant proteins, fatty acids, refined grains, sodium, added sugars and saturated fats (Krebs-Smith et al., 2018a). It has been shown that there is a relationship between the HEI scores and the development of some types of cancer (Chandran et al., 2011; Arem et al., 2013; Shahril et al., 2013). Individuals with a high score in HEI are generally less prone to cancer development (Seymour et al., 2003). However, to date, there has been no study evaluating the association of the HEI-2015 scores with breast cancer risk. Therefore, the present study was undertaken to evaluate the association between the HEI-2015 scores and breast cancer risk.

## Materials and Methods

*Study design*

A case-control study involving 134 women with BC and 265 cancer-free controls were conducted. Cases were women aged 30-65 years, who were histologically confirmed as having BC (ductal carcinoma) within 5 months from the recruitment date. All cases were recruited from the general hospital of Tehran, Iran. Patients with a history of any cyst or cancer (except the current BC) and those who were under hormone replacement therapy were excluded. Controls were age-matched women recruited from other wards of the same hospital with no history of cysts, cancer, or hormone replacement therapy. All participants (cases and controls) were excluded from the study if their reported energy intake was ≤ 500 Kcal/day or ≥ 5,000 Kcal/day. In addition, participants who skipped ≥ 50 items in the food frequency questionnaire (FFQ) were excluded from the study. Nine participants from the control group withdrew from the study and one was excluded due to the incomplete food item records. All participants signed a written informed consent form before enrolment into the study. The study protocol was approved by the Research Institute for Nutrition and Food Sciences, Shahid Beheshti University of Medical Sciences.

*Dietary assessment*

The dietary records of the participants were collected by trained dietitian interviewers. A validated FFQ consisting of 168 items was used to collect dietary information semi-quantitatively (Mirmiran et al., 2010). The interviewers recorded dietary intake of each food item on the basis of daily, weekly and monthly frequency. The portion sizes of selected food items were determined based on the US Department of Agriculture portion sizes and household measures and were then converted to grams (Ghafarpour et al., 1999). 

*Healthy Eating Index-2015 (HEI-2015) score calculation*

The HEI-2015 comprised 13 components, namely 1) Total Fruits; 2) Whole Fruits, 3) Total Vegetables; 4) Greens and Beans; 5) Total Protein Foods; 6) Seafood and Plant Proteins; 7) Whole Grains; 8) Dairy; 9) Fatty Acids; 10) Refined Grains; 11) Sodium; 12) Added Sugars; and 13) Saturated Fats. The first six items carried a maximum of five points each while the other items carried a maximum of ten points each. In order to calculate the scores of the HEI-2015, amounts consumed from each food item were acquired from FFQ and food groups were converted to cup and ounce equivalents. The ‘Total Protein Foods’ consisted of servings of meat, poultry, seafood, eggs, legumes, seeds, nuts (beans and peas) and soy products. The ‘Total Fruits’ component included servings of whole fruit and fruit juice. The ‘Seafood and Plant Proteins’ included servings of seafood, seeds, nuts, legumes (beans and peas) and soy products. The ‘Whole Grain Foods’ consisted of servings of legumes (beans and peas) and dark-green vegetables while the ‘Total Vegetables’ comprised servings of legumes (beans and peas), dark-green vegetables, and all other vegetables. The ‘Fatty Acids’ was considered as a ratio of polyunsaturated (PUFA) and monounsaturated fatty acids (MUFA) to saturated fatty acids (SFA). The ‘Saturated Fats’ and ‘Added Sugars’ were transformed to the percent of total energy intake and the rest of the food ingredients were transformed to the amounts per 1,000 kcal except for Fatty Acids. The range of the HEI-2015 score was between 0-100.

*Measurements*

The weight of the participants was measured to the nearest 0.1 kg using a digital scale (Model 803; Seca, Hamburg, Germany) on a flat and uncarpeted surface with minimum clothes and without shoes. The height was determined using a stadiometer (Model 206 Portable Body Meter Measuring Device; Seca) to the nearest 0.5 cm without shoes. The BMI was calculated by dividing weight in kg to height squared in meters. The level of physical activity during the past year was determined using a validated questionnaire (IPAQ) during the interview and was expressed as metabolic equivalent hours per day (MET-h/day).

*Statistical analysis*

In order to determine the difference in general characteristics between cases and controls, analysis of variance (ANOVA) and chi-square tests were used for continuous and categorical variables, respectively. The participants were assigned to a quartile according to their scores on HEI-2015. The means of age, weight, height, BMI, energy intake and HEI scores were specified across the quartiles using the general linear model. 

The association between the HEI scores and breast cancer risk was determined using multivariable logistic regression models across the quartiles. The model was adjusted for confounding variables such as age (y); BMI (kg/m2); educational level (y); occupation (housekeeper/ employee/ retired); use of alcohol and tobacco (yes/no); age at menarche (y); marital status (not married, married, divorced, widow); age at first pregnancy (y); number of full pregnancy; menopause status (yes/no); family history of breast cancer (yes/ no); use of OCP (yes/no); use of bra (<12h, >12h); life satisfaction (yes/no/partly); physical activity (MET-h/ week); energy intake (kcal/d); energy density of diet (kcal/100g foods).

In order to determine the trend of the association across specified quartiles of the HEI scores, the median of each quartile was used as a continuous variable in the logistic regression model. All statistical analyses were performed using SPSS (version 16.0, IBM Co., Chicago, IL). A p-value of <0.05 was considered statistically significant. 

## Results

General characteristics of the cases and controls are shown in Table 1. The mean age of the cases was significantly higher compared with the controls (49.49 ±10.67 vs. 47.13 ± 10.08, P=0.031). No statistically significant differences were observed for the other variables.

Usual intakes of selected food and nutrient groups in cases and controls are provided in Table 2. Cases had significantly lower intakes of energy, proteins, carbohydrates, total dietary fiber, vitamin B9, selenium, zinc, magnesium, iron, and calcium than the controls (P<0.05). For the other food and nutrient groups, a significant difference was not observed between the two groups of participants. 

The association between the HEI-2015 scores and breast cancer risk is summarized in Table 3. In the crude model, subjects in the top quartile of the HEI-2015 scores had a 46% lower chance of breast cancer compared to subjects in the bottom quartile (OR 0.54; 95% CI 0.30, 0.98). After adjustment for potential confounders such as age, age at menarche, oral contraceptive drug use, menopausal status, marital status, body mass index, smoking and education level, this association was enhanced (OR 0.32; 95% CI 0.16, 0.65).

**Table 1:** Selected Characteristics of Cases and Controls Participating in the Study^a^

Characteristic	Cases (n = 134)	Controls (n = 265)	*P*-Value^b^
Age	49.49 ±10.67	47.13 ± 10.08	0.031
Height (cm)	157.84± 6.11	159.11 ± 6.26	0.053
Weight (kg)	73.05±14.31	73.39±12.95	0.258
BMI^c^	30.15± 5.67	29.07± 5.39	0.066
Waist (cm)	99.50±14.52	96.39±13.25	0.076
Marital status			
Single	9 (56.5)	16 (6)	0.929
married	105 (43.5)	206 (77.4)	
divorced	5 (3.8)	13 (4.9)	
widowed	14 (10.5)	31 (11.7)	
Education			
Primary	13 (10)	24 (9)	0.316
Secondary and high school	55 (42.3)	134 (50.4)	
University	62 (47.7)	108 (40.6)	
Smoking	4 (3)	9 (3.4)	0.842

a, Data are presented as mean ± SD or n (%); b, Independent samples t-test was used for continuous variables and Chi-square test was used for categorical variables; c, Body Mass Index (kg/m^2^)

**Table 2:** Distribution of Dietary Intakes of Macro and Micronutrients in Cases and Controlsa

Characteristic	Cases (n = 62)	Controls (n = 124)	*P*-Value^b^
Total calories (kcal/day)	2562.57±612.84	2753.45±798.02	0.008
Total protein intake (g/day)	80.84±23.49	88.47±25.61	0.004
Total carbohydrate intake (g/day)	341.62±80.50	372.54±114.67	0.002
Total fat (g/day)	103.70 ±36.24	108.24 ±41.78	0.284
Total fiber(g/day)	36.54±14.34	39.89±18.58	0.047
Cholesterol (mg/day)	284.67 ±142.99	293.52±135.55	0.553
SAFAs (g/day)	31.76 ±9.73	32.92 ±11.26	0.287
MUFAs (g/day)	36.61 ±14.13	37.24 ±15.97	0.687
PUFAs (g/day)	24.07 ±11.43	24.49 ±13.29	0.746
Trans-fatty acids (g/day)	0.145±0.303	0.154 ±0.265	0.785
Vitamin B6 (mg/day)	1.80±0.54	1.95±0.55	0.12
Vitamin B9 (ug/day)	413.94±125.30	455.20±163.07	0.005
selenium (mg/day)	92.96±37.18	101.39±41.07	0.04
Sodium (mg/day)	4521.44±1503.90	4740.74±1811.45	0.2
Zinc (mg/day)	11.87±3.86	12.95±4.059	0.01
Copper (ug/day)	1.74±0.63	1.85±0.72	0.116
Magnesium (mg/day)	364.42±124.67	402.91±133.15	0.005
Iron (mg/day)	14.92±4.94	16.34±6.06	0.012
Calcium (mg/day)	1220.93±465.41	1335.27±458.76	0.02

a, Data are presented as mean ± SD; b, Obtained from ANCOVA. all values except energy intake are adjusted for age, gender and energy intake.SFAs, Saturated fatty acids; MUFA, mono-unsaturated fatty acids; PUFA, polyunsaturated fatty acids; EPA, Eicosapentaenoic acid; DHA, Docosahexaenoic acid.

**Table 3:** Odds Ratios and Confidence Intervals for the Association between Dietary Total Antioxidant Capacity and Ulcerative Colitis

	Quartile1	Quartile2	Quartile3	Quartile4	*P*-value for Trend
	<72.73	72.74-76.25	76.26-79.93	≤79.94	
Case/control	42/59	36/64	28/72	28/72	-
Crude	Ref	0.79 (0.44-1.39)	0.54 (0.30-0.98)	0.54 (0.30-0.98)	0.021
Model 1^a^	Ref	0.73 (0.41-1.31)	0.52 (0.29-0.95)	0.47 (0.26-0.87)	0.008
Model 2^b^	Ref	0.63 (0.33-1.119)	0.50 (0.27-1.03)	0.32 (0.16-0.65)	0.001

a, Adjusted for age; b, Adjusted for age, age at menarche, oral contraceptive drug use, menopausal status, material status, body mass index, smoking, education.

## Discussion

Over the past decades, nutritional epidemiological studies have identified a number of food or nutrients which could affect the risk of breast cancer, but the results remain inconsistent (Takagi et al., 2015; Messina, 2016; O’Brien et al., 2017). One explanation for the inconsistency in the study findings was that the effect of a single food or nutrient could be easily reversed by another food or nutrient (Tapsell et al., 2016). Thus, modern nutritional epidemiological studies have focused on assessing the effect of the overall diet quality in order to better capture the dietary variability in the population. The Healthy Eating Index (HEI) is one of the most commonly used measures of the overall diet quality (Krebs-Smith et al., 2018b). In this work, we investigated the association between the HEI-2015 scores and breast cancer risk. A high HEI-2015 score indicated a high intake of fruits and vegetables, whole grain, dairy products, protein and unsaturated fatty acids, while a low score indicated a high intake of refined grains, sodium, added sugars and saturated fats (Krebs-Smith et al., 2018b). We observed that high HEI-2015 scores were significantly associated with a decreased risk of breast cancer. This association was strengthened after adjustment for various risk factors of breast cancer, suggesting that the observation was robust. 

Fruits, vegetables and whole-grain are rich in bioactive substances such as vitamins and carotenoids which possess antioxidant properties; thus, it is not surprising that a high intake of these foods can prevent cellular damage by free radicals and lead to a reduction in breast cancer risk (Saini et al., 2015). In addition, fruits, vegetables and whole-grain have a high content of dietary fiber and other nutrients that can reduce the levels of estrogen and N-nitroso compounds as well as increase the levels of anti-inflammatory cytokines, which collectively prevent breast cancer initiation and progression (Saini et al., 2015). On the other hand, dairy products are double-edged swords in regard to their effect on cancer risk. While some evidence suggested that dairy products contain high amounts of calcium and vitamin D which can bind to cancer-causing acids and metabolites to reduce breast cancer risk (McCann et al., 2017), some other evidence found that dairy products may increase cell proliferation due to their high levels of growth-promoting factors such as insulin-like growth factor 1 (IGF-1) (Djamil and Arezki, 2015). Besides, unsaturated fatty acids can decrease the levels of free radicals and alter estrogen metabolism, which protects against cancer development (Khodarahmi and Azadbakht, 2014). These observations explain why a higher HEI-2015 score was associated with a lower risk of breast cancer as noted in the present work.

There are a few strengths and limitations of the present study. A major limitation was that there could be recall bias among the participants as the study was retrospective in design (Althubaiti, 2016). However, the recall bias is likely minimal as the dietary data was collected by trained dietitian interviewers using a validated FFQ. Another limitation of the present study was the modest sample size. Despite this, the study has generated important data that could serve as a solid foundation for future research in this area, which represents one of the major strengths of this study. In fact, to the best of our knowledge, this is the first study that investigated the association between HEI-2015 scores and breast cancer risk. In addition, we adjusted our study findings for many potential risk factors of breast cancer, which ensured that our study finding was not affected by potential confounders.

In conclusion, the present work has successfully shown that a high HEI-2015 score was associated with a reduced risk of breast cancer. Our finding suggests that an overall healthy diet is important for the prevention of breast cancer; thus, a diet rich in fruit, vegetables, whole grains, dairy products and unsaturated fats should be encouraged. Nonetheless, future studies, especially prospective cohort studies, are warranted to confirm our findings.
